# The National Medical Union (Official)

**Published:** 1914-06-06

**Authors:** V. T. Greenyer, Robert Carswell, E. Arthur Dorrell

**Affiliations:** Chairman of Council; Honorary Secretary; Honorary Secretary


### THE NATIONAL MEDICAL UNION (Official).

Offices: 346 Strand. Tel.: City 8444.

### HONORARY OFFICERS.

**Presidents.**

G. A. Wright, F.R.C.S. (Manchester).
Professor Wm. Russell (Edinburgh).

**Vice-Presidents.**

Wm. Coates, C.B., D.L. (Manchester).
V. T. Greenyer, F.R.C.S. (Brighton).
John Playfair, M.D. (Edinburgh).

**Honorary Secretaries.**

Robert Carswell, M.A., M.B. (London).
E. Arthur Dorrell, F.R.C.S. (London).

**Honorary Treasurers.**

H. Oppenheimer, M.D., LL.D. (London), 19 College Crescent, Hampstead.
J. Brassey Brierley, M.D. (Manchester), 522 Stretford Road, Old Trafford.

### PROVISIONAL COUNCIL.

J. Ford Anderson, M.D. (London).
J. L. Baskin, M.D. (Brighton).
A. Bousfield, M.D. (London).
W. F. R. Burgess, M.D. (London).
H. B. Greene, M.R.C.S. (London).
Francis Heatherley, F.R.C.S. (Liverpool).
L. F. Hemmans, M.B., B.S., F.R.C.S.Edin. (London).
T. Johnston, M.R.C.S. (Brighton).
S. Lee, L.R.C.P.I. (London).
C. E. M. Lowe, M.B. (Crewe).
J. T. Macnamara, L.R.C.P.I. (London).
G. H. Pinder, M.R.C.S. (Manchester).
Frederick Porter, M.B. (Edinburgh).
J. Skardon Prowse, M.B. (Manchester).
Henry Sharman, M.D. (London).
A. F. Shoyer, M.D. (London).
James Smith, M.D. (Edinburgh).
F. W. Spurgin, M.R.C.S. (London).
W. Percy Stocks, F.R.C.S. (Manchester).
T. H. Thyne, M.B., F.R.C.P. (Edinburgh).
F. H. Westmacott, F.R.C.S. (Manchester).
G. D. Wilson, L.R.C.P. (London).

The National Medical Union was formed at a Conference of Delegates from Non-Panel Associations, representing over 1,000 members, held in London on November 29, 1913. At this Conference it was unanimously resolved to form a Federation of Non-Panel Associations, of which the primary condition of membership should be that applicants for membership should not be on the panel directly or indirectly. The title "National Medical Union" was taken over, by consent, from the body of the same name formed at Manchester in 1912, which had taken the leading part in organising the Conference. The Union thus formed marks an important stage in the development of medical organisation, embodying the characteristics of its main sources of origin—namely, the National Medical Union of Manchester, the Edinburgh Medical Guild, and the London Medical Committee and Non-Panel Associations.

The Manchester Union, formed at Manchester in 1912, with members all over the country, adopted at the very outset the uncompromising policy of "no service whatever" under the Insurance Act, a policy which it steadfastly maintained throughout. In October 1913, acting on information supplied by the Wandsworth Non-Panel Association, the Manchester Union decided to summon the Conference which resulted in the successful establishment of the new Union.

The Edinburgh Medical Guild, a contemporary of the Manchester Union, has been characterised by its opposition to "contract practice," by its defence of the honourable ideals and traditions of the profession, and by the chivalrous way in which the leaders of the profession in Edinburgh have supported by their active participation the efforts of the general practitioners in defence of their freedom and principles.

In London a remarkable fight was put up by the London Medical Committee, which organised the profession in the London area to such good purpose that after the lapse of a year of the working of the Act it was found that only 1,468 out of a total of 6,606 London practitioners were found to have joined the panels, in spite of special inducements in the shape of "secret" panels and two varieties of special contract forms being offered to them. Another gratifying result upon which the London Medical Committee may plume themselves is the large number of insured persons who have remained with their own doctors in preference to choosing one from the panel.

### REPORT OF PROVISIONAL FEDERATING COUNCIL.

At the Conference at which the National Medical Union was formed, a Provisional Council was elected for the purpose of (1) carrying out the federation of associations and enrolling new members; (2) taking and furnishing suitable offices, together with engaging necessary clerical assistance; (3) drafting proposals for a constitution; (4) making proposals for a policy; (5) dealing with any other matters of an urgent nature.

The Council has now completed its work and begs to report as follows:—

### (1) Federation of Societies.

Of the societies represented at the Conference the following thirteen have joined the Federation: 1, Crystal Palace; 2, Deptford, Greenwich, and Camberwell; 3, Edinburgh; 4, Hampstead; 5, Huddersfield; 6, Islington and St. Pancras; 7, Lambeth; 8, Lewisham; 9, Liverpool; 10, Manchester; 11, Sussex; 12, Wandsworth; 13, Willesden. Also a number of isolated members in various parts of the country have been enrolled pending the formation of associations in their own neighbourhood.

The work of promoting new associations and enrolling members has been put into the hands of an Organisation Committee which has arranged that Scotland should be worked from Edinburgh, the North of England from Manchester, and London and the South of England from the head office.

In addition to giving as much publicity in the press as was possible to the existence of the Union, the Council has collected a complete set of panel lists for counties and county boroughs in England, and by deleting these names from the Medical Directory and Medical Register has now in its possession a register of non-panel doctors in England.

The enrolment of members has proceeded most actively in London for various reasons. The London list of Churchill's Directory, cleared of panel names, has been divided up into alphabetical lists for each borough, or group of boroughs, and these lists have been put into the hands of secretaries of local associations, or, in certain instances, of unattached members, for the purpose of securing a systematic canvass and classification. Elsewhere the work of organisation has not yet proceeded so far, but it will follow similar lines.

### (2) Offices and Clerical Assistance.

The Council has secured offices in a central position at 346 Strand, on a three years' agreement, terminable at shorter intervals, at a rental of £90 per annum. The services of a shorthand typist have been engaged, together with other temporary assistance.

### (3) Draft Constitution.

The Council recommends: (1) That the National Medical Union should apply for registration under the Companies Act, 1867, and that the liability of members should be limited by guarantee to £1. (2) That the permanent officers should be two honorary presidents, not less than three honorary vice-presidents, secretaries, treasurers, and Council, with committees—executive, organising, finance, Parliamentary, and press. (3) That immediately after the general meeting the local associations should themselves conduct an election for the membership of the first permanent Council. (4) Every association of twenty-five subscribers and under should be represented by one member of Council, with an additional member for every additional twenty subscribers, the minimum membership entitling any society to elect a member of Council being fixed at ten; associations having under ten members to elect their representatives in common with the unattached members of the Union through the central office. (5) That the chairman of Council, together with the secretaries, should be *ex officio* members of all committees, and that the following permanent committees should form part of the constitution:—(*a*) An Executive Committee, consisting of twelve members, five *ex officio*, including the hon. treasurers, seven elected, the duties of this committee being to carry out decisions of the Council and in emergencies to exercise all the functions vested in the Council. (*b*) An Organising Committee of twelve members, three *ex officio*, nine elected. (*c*) A Finance Committee of eight members, five *ex officio*, including the hon. treasurers. (*d*) A Parliamentary Committee of eight members, three *ex officio*, five elected. (*e*) A Press Committee of six members, three *ex officio*, and three elected.

The Council is of opinion that as soon as funds permit the services of a paid business manager should be secured.

(4) RESOLUTIONS ON POLICY.

The following resolutions on policy have been adopted by the Council for recommendation to the general meeting:—That it be the policy of the National Medical Union—

1. To organise the medical profession to the highest possible degree of efficiency in defence of its traditional freedom and in protection of its economic interests.

2. To render every service possible to non-panel practitioners in their unequal struggle against the difficulties brought about by acceptation by panel practitioners of service under the National Insurance Acts.

3. To oppose any prospective legislation to include dependants of insured persons participating in the medical benefit of the Acts under the present arrangements.

4. To agitate for repeal of the Medical Benefit Sections of the Insurance Acts, or such drastic amendments as would bring these sections into harmony with the principles of freedom on the part of the profession and public as already laid down by the Union.

5. To lay down the principle of unrestricted free choice of doctor in any form of National Insurance, and to insist upon the unrestricted right of insured persons to make individually their own arrangements in connection with the Insurance Acts, 1911-1913; and, further, to insist that no doctor attending such persons be required to sign any contract with the Commissioners or any Committee under the Acts.

6. To place before the public in any and every way possible the dangers of the Acts to public health, their injustice to doctor and patient alike, and the iniquity of continuing to work them under the present conditions.

7. To maintain that a medical service is necessary for the genuinely poor classes of the community *and for them only.*

8. To preserve an independent attitude with regard to party politics.

The National Medical Union is not opposed to the principle of voluntary Health Insurance under such conditions as would conform with the other principles of its policy.

FORM 43 I.C.

The question of the signing of Form 43 I.C., or any similar form in relation to any patient for whom a medical man has not already signed such form, is referred by the Council to the general meeting with the unanimous expression of opinion on the part of the Council that no member should sign this form or any similar form.

SEAMEN'S NATIONAL INSURANCE SOCIETY.

The Council is of opinion that practitioners attending members of the Seamen's National Insurance Society should not be debarred from belonging to the National Medical Union, but the Council of the National Medical Union recommend practitioners not to sign the agreement of the Seamen's National Insurance Society.

In the course of its term of office the Council took exception to Regulation 44 (2) of the Insurance Commissioners authorising unqualified practice, issued a public protest, and drew the attention of the General Medical Council to the subject. Similar protests were subsequently issued by the Royal College of Physicians, the Royal College of Surgeons, and the Society of Apothecaries.

A useful step was taken by the Union in connection with a proposed Statutory Local Medical Committee for London, which was to be set up on an entirely unsatisfactory basis, and with a view to giving an appearance of support by the medical profession in London to the National Insurance Act which the latter does not possess. The Union promptly circularised the profession to this effect, with the result that nearly every nomination representative of non-panel practitioners was withdrawn.

V. T. GREENYER,
Chairman of Council.
ROBERT CARSWELL,
E. ARTHUR DORRELL,
Honorary Secretaries.

**NATIONAL MEDICAL UNION DINNER.**

All particulars of the date, place, etc., of the National Medical Union dinner will be found in a Note on page 255.

